# Questions and controversies in innate immune research: what is the physiological role of NLRP3?

**DOI:** 10.1038/cddiscovery.2016.19

**Published:** 2016-04-04

**Authors:** RC Coll, LAJ O’Neill, K Schroder

**Affiliations:** 1 Institute for Molecular Bioscience (IMB), IMB Centre for Inflammation and Disease Research and Australian Infectious Diseases Research Centre, The University of Queensland, Brisbane, St Lucia 4072, Australia; 2 School of Biochemistry and Immunology, Trinity Biomedical Sciences Institute, Trinity College Dublin, Dublin, Ireland

## Abstract

The NLRP3 inflammasome is a key component of the innate immune system that induces pro-inflammatory cytokine production and cell death. Although NLRP3 is activated by many pathogens, it only appears to be critical for host defense for a limited number of specific infections. NLRP3 is however strongly associated with the initiation and pathology of many inflammatory diseases. If NLRP3 function is largely redundant for host defense, but drives a number of inflammatory diseases, this raises the important question of why evolution has elected to maintain NLRP3 function. We propose that the primary physiological functions of NLRP3 in health are to engage pathways to clear noxious substances (e.g. protein aggregates and crystals), and to regulate metabolism. We discuss the newly identified functions for NLRP3 in metabolic homeostasis, and how NLRP3 beneficial functions in homeostasis may become detrimental during the onset of inflammatory and metabolic diseases. A common feature of most NLRP3-driven diseases is that they are associated with ageing or metabolic excess, and indeed, *Nlrp3* deficiency promotes ‘healthspan’ in ageing mice. This suggests that beneficial functions of NLRP3 in youth may become increasingly countered by NLRP3-dependent pathology as an individual ages, and we propose a general model by which ageing or nutrient excess may provide a tipping point to switch NLRP3 function from beneficial to pathological. The physiological role of NLRP3 in healthy individuals remains incompletely understood and future research will need to address this if NLRP3 is to become a successful therapeutic target for the clinical management of inflammatory diseases.

## Facts

- Nod-like receptor protein 3 (NLRP3) is a cytosolic pattern recognition receptor that is activated by a wide range of pathogen-, host- and environment-derived molecules.
- Active NLRP3 forms a large protein complex called the inflammasome that mediates caspase-1 activation.
- Caspase-1 cleaves the pro-inflammatory cytokines interleukin (IL)-1*β* and IL-18 to their active secreted forms and also causes a form of lytic inflammatory cell death known as pyroptosis.
- Aberrant NLRP3 activation is associated with many human inflammatory diseases and is an emerging target for drug development.

## Open questions

- Why is NLRP3 associated with so many inflammatory diseases and how did this situation evolve?
- Is NLRP3 function critical for host defense?
- Does NLRP3 maintain metabolic homeostasis in healthy individuals?
- What are the IL-1-independent functions of NLRP3?
- Can NLRP3 be safely targeted for inflammatory disease treatment?

**Figure 1:**
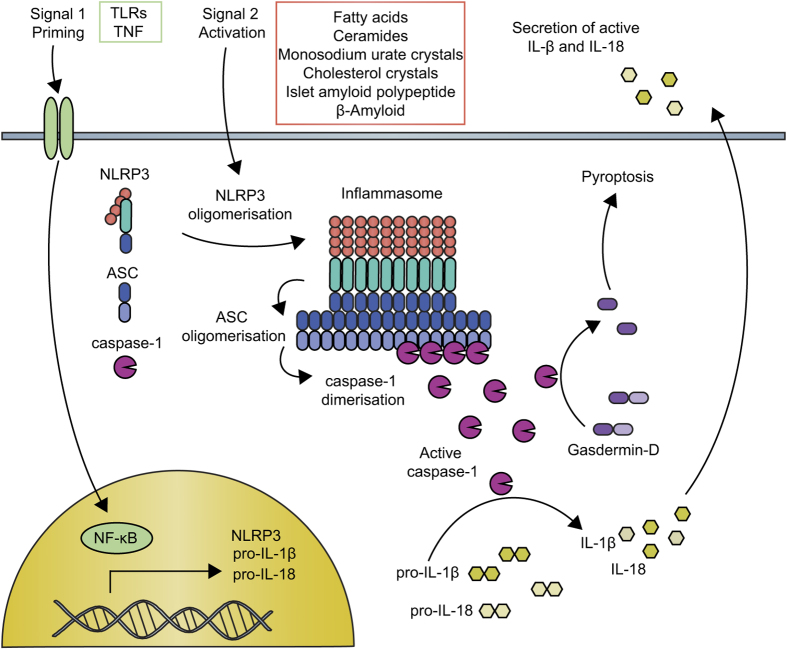
The NLRP3 inflammasome signaling pathway. NLRP3 inflammasome activation requires an initial ‘signal 1’, such as toll-like receptor or TNF receptor activation, that activates NF-κB. This priming signal induces the transcription of NLRP3 and pro-IL-1*β* and also results in post-translational modification of the NLRP3 protein, which is required for activation. A second NLRP3-specific activation signal (‘signal 2’, such as the metabolic substances indicated) triggers conformational changes resulting in the oligomerization of NLRP3. The adapter molecule ASC is recruited through Pyrin domain interactions with NLRP3 and forms large prion-like oligomers. The ASC CARD domain interacts with caspase-1 and ASC oligomers provide a scaffold for caspase-1 dimerization, autocatalytic processing and the generation of active caspase-1. Active caspase-1 processes pro-IL-1*β* and pro-IL-18 to their active mature forms, which are secreted. Caspase-1 also cleaves Gasdermin-D resulting in inflammatory cell death (pyroptosis).

In 2001, nod-like receptor protein 3 (NLRP3) mutation was discovered to cause a group of rare inherited diseases characterized by recurrent episodes of inflammation.^1^ Since then, we have learned that NLRP3 initiates pro-inflammatory signaling through the formation of a protein complex called the inflammasome, and disease-driving mutations potentiate this pathway.^2^ Activation of NLRP3 triggers its oligomerisation, which in turn allows for the recruitment and clustering of the ASC inflammasome adapter and the zymogen protease, caspase-1. Caspase-1 is then activated, and cleaves the interleukins (ILs)-1*β* and -18 to generate their active, secreted forms. Caspase-1 also initiates a form of cell death called pyroptosis. The NLRP3 inflammasome signaling pathway is illustrated in Figure 1. A number of other NLRs and pattern recognition receptors (PRRs) can also form inflammasomes, the best studied of these being NLRP1, NLRC4 and AIM2.^2^ Although most PRRs are activated by direct interaction with well-defined ligands, NLRP3 appears to be unique in both the number and diversity of substances that it can sense. These include microbial, environmental and host-derived factors such as crystals, protein aggregates, pore-forming toxins, nucleic acids and ATP. NLRP3 can thus be regarded as a broad sensor of ‘danger’ or the disruption of cellular homeostasis, although the precise mechanism(s) by which NLRP3 senses homeostatic disturbance remain obscure.^3^

Although NLRP3 is activated by a wide variety of pathogens including bacteria, viruses, protozoa and fungi, it does not appear to be critical for host defense for many infections *in vivo*.^4,5^ This may be due to redundancy with other inflammasomes, as a number of pathogens can induce caspase-1 activation and IL-1*β* release through multiple inflammasome sensors. For example, both NLRP3 and AIM2 provide resistance in *Aspergillus* infection,^6^ and NLRP3 and NLRP1 are both required for survival during *Toxoplasma gondii* infection.^7^ For Gram-negative bacteria, deficiency in either *Nlrp3* or *Nlrc4* renders mice more susceptible to *Burkholderia pseudomallei,* and NLRP3, NLRC4 and caspase-11 together provide host defense against *Salmonella typhimurium*.^8,9^ Redundancy between inflammasomes may have evolved to combat microbial subversion of these sensors.^10^

Although NLRP3 function appears somewhat redundant during infection, compelling evidence supports a non-redundant, pathological role of NLRP3 in a large number of inflammatory diseases including metabolic syndrome and type 2 diabetes, non-alcoholic fatty liver disease/non-alcoholic steatohepatitis, gout, atherosclerosis, multiple sclerosis, Alzheimer’s disease and Parkinson’s disease.^11^ *Nlrp3-*deficient mice housed under standard conditions are generally healthy.^12^ If NLRP3 is not essential for health, and indeed may be detrimental, this raises the question of what physiological function NLRP3 fulfills in healthy individuals. The established role of NLRP3 as an inflammation initiator during homeostatic disturbance suggests that the physiological role of NLRP3 may be to restore homeostasis during day-to-day fluctuations in the cellular environment.^13^ The clear contribution of NLRP3 to many inflammatory diseases implies that such a proposed role for NLRP3 only operates within strict boundaries that are surpassed during disease onset. In this scenario, large or chronic homeostatic fluctuations would switch the NLRP3 pathway from beneficial to pathological. Such a pathological role may avoid the negative selection of *Nlrp3* during evolution, since most NLRP3-driven pathologies are associated with ageing and so may not compromise reproductive success. NLRP3 may in fact only be pathologic when the concentration of the offending substance reaches a certain threshold, the build up (e.g. for factors such as *β*-amyloid and cholesterol crystals) being age-related. Otherwise NLRP3 can promote clearance of the insult without being detrimental, as might apply during reproductive years. Here we briefly discuss possible beneficial functions for NLRP3 in maintaining health, and how these functions may be become pathological in disease.

## NLRP3 in immunometabolism

NLRP3 is activated by numerous substances indicative of metabolic surplus, such as saturated fatty acids,^14^ ceramides,^15^ monosodium urate^16^ and cholesterol crystals.^17^ NLRP3 is important for driving localized inflammation in response to noxious substances such as protein aggregates (e.g. islet amyloid polypeptide)^18^ and crystals (e.g. monosodium urate crystals). NLRP3-dependent IL-1*β* potently recruits phagocytes that can digest and remove these harmful particles.^16^ Thus, NLRP3-dependent pathways may assist in the removal of noxious substances.

NLRP3 may also be required for regulating energy metabolism (extensively reviewed in ref. 13), but here the role of NLRP3 is more difficult to decipher as it can influence pro-inflammatory cytokine production (e.g. IL-1*β*) and cleavage of other caspase-1 substrates in multiple cell types (e.g. hematopoetic and non-hematopoetic) and tissues (e.g. pancreas and adipose). IL-1*β* signaling has both local and systemic effects that contribute to regulating energy homeostasis.^19^ For example, low levels of IL-1*β* in the pancreas can induce *β*-cell proliferation^20^ and insulin release,^20,21^ and so contribute to the restoration of normoglycemia. Early studies with systemically administered IL-1 demonstrated that it induced anorexia.^22^ Fatty acids trigger NLRP3 responses,^14^ NLRP3 regulates triglyceride metabolism via an IL-1*β*-independent mechanism,^23^ and indeed, *Nlrp3*-deficient mice show accelerated clearance of triglycerides after a fat challenge.^23^ Interestingly, NLRP3 functions in steady-state lipid metabolism were mediated by non-hematopoetic cells.^23^ In all, these studies suggest that NLRP3 regulates glucose and triglyceride metabolism during the normal metabolic fluctuations that occur each day with nutrient intake and energy expenditure in healthy individuals. Surprisingly, however, mice deficient in *Nlrp3*, *Asc* or *Il1r1* fed a normal chow diet do not show any gross changes in adiposity compared with wild-type mice.^12^ This is in contrast to *Il18*- and *Il18r*-deficient mice that are hyperphagic, and become spontaneously obese and insulin resistant.^24^ In this context, the predominant inflammasome controlling IL-18 appears to be NLRP1, as *Nlrp1*-deficient mice also become spontaneously obese.^25^ This suggests that while NLRP3 activation can trigger IL-18 secretion, NLRP3-regulated IL-18 is largely dispensable for metabolic homeostasis in health.

In addition to nutrient surplus, the initiation of metabolic disease is also associated with mitochondrial dysfunction.^26^ NLRP3 is a proposed sensor for mitochondrial damage and dysfunction through direct interaction with mitochondrial DNA and cardiolipin, or indirectly, through sensing reactive oxygen species generated by dysfunctional mitochondria.^11^ In keeping with a disease-driving role for NLRP3 activated by nutrient excess and mitochondrial dysfunction, *Nlrp3*-deficient mice appear to be resistant to several metabolic diseases. For example, in murine high-fat diet models, deficiency in *Nlrp3*, *Casp1/11*, *Asc*, *Il1b* or *Il1r* protects mice from developing obesity and insulin resistance.^14,15,27,28^ Mechanistically, increased levels of IL-1*β* inhibits insulin signaling in adipocytes and hepatocytes, and induces pancreatic *β*-cell dysfunction.^13^ So while NLRP3 and IL-1*β* may perform beneficial functions in metabolic regulation in the steady-state, NLRP3 appears to respond aberrantly to excess, in this case excessive nutrients and/or dysfunctional mitochondria.

Overall these data suggest that in healthy individuals NLRP3 may be required for sensing metabolic disturbance and initiating mechanisms that return the system to homeostasis. However, in the case of chronic exposure to metabolic surplus, the homeostatic threshold of NLRP3 may be surpassed, resulting in excessive and detrimental inflammation, and ultimately disease. Metabolic diseases such as type 2 diabetes are generally lifestyle-associated, and develop in middle to old age, and as such have little impact on reproductive fitness. NLRP3 may thus be an example of antagonistic pleiotropy where this gene controls traits that are both beneficial and detrimental to the organism.^29^

## Other potential NLRP3 homeostatic functions

IL-1 causes a large number of local and systemic biological effects that have been well characterized over decades.^19^ As NLRP3 characterization is still ongoing, evidence specifically linking NLRP3 to many of these IL-1-mediated processes is still lacking in many cases. For example, IL-1 is a well-established driver of hematopoesis in response to infection or myelosupression,^19^ but potential roles for NLRP3 in steady-state or emergency hematopoesis have not yet been explored in detail. A number of recent studies have identified IL-1*β*-independent functions for NLRP3, which is not unexpected as caspase-1 cleaves cellular substrates other than IL-1*β* and IL-18.^30^ A number of such examples are discussed below:

### Aging

One of the most intriguing phenotypes observed in aged (i.e. >18 months) *Nlrp3*-deficient mice is their prolonged healthspan.^12^ The age-related decline in glucose tolerance, bone density and thymic mass observed in mice appears to require NLRP3 but not IL-1 signaling, while the NLRP3/IL-1 axis was required for age-related cognitive decline associated with central nervous system inflammation.^12^

### Microbiome

A single study comparing naïve wild type and *Nlrp3* ^−/−^ littermates found there were 10 bacterial genera clearly enriched in the fecal microbiota of *Nlrp3-*deficient animals.^31^ Currently, the function of NLRP3 in the intestine remains controversial with reports of both protective and deleterious effects of NLRP3, particularly in murine models of colitis.^32^ Additional carefully controlled studies to examine NLRP3 pathways in intestinal homeostasis are clearly required.

### Transcription

A recent article by Bruchard *et al.* ^33^ demonstrated that NLRP3 binds to the IL-4 promoter in CD4^+^ T cells. In this context, NLRP3 functioned independently of inflammasomes as a transcriptional regulator to influence Th2 cell differentiation.^33^ Thus, inflammasome-independent NLRP3 may influence adaptive immunity and Th2 responses.

**Figure 2:**
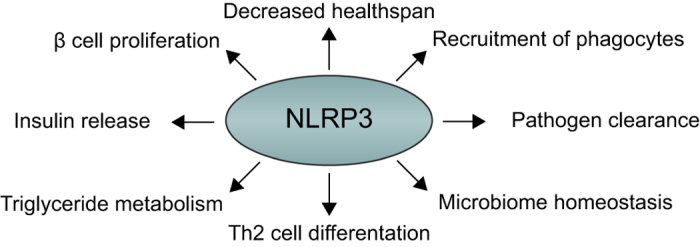
Positive regulatory functions for NLRP3 in homeostasis. NLRP3 has a number of functions in metabolism such as regulating insulin release, *β*-cell proliferation and triglyceride levels. New IL-1*β*-independent functions of NLRP3 have been identified in regulating ageing, intestinal microbiota and during transcription in Th2 cells. NLRP3 is required for the sensing of pathogens such as *Candida* and in the recruitment of phagocytes to clear infections and noxious protein aggregates and crystals.

A summary of the current knowledge of the homeostatic functions of NLRP3 is shown in Figure 2.

## Considerations for NLRP3 targeting in disease

We recently characterized an NLRP3 small molecule inhibitor called MCC950.^34^ Our data suggests that NLRP3 inhibition by a small molecule may offer a new, more-targeted approach for treating NLRP3-driven diseases than current biologics that block IL-1 signaling. Given the above discussion of possible beneficial, homeostatic NLRP3 functions, the potential detrimental effects of NLRP3 inhibition should also be considered. For example, could such therapy increase a patient’s risk of infection? IL-1 blockade in patients does cause a small increase in the incidence of infections.^35^ However, the redundancy within and between IL-1-producing inflammasomes and other PRR pathways suggests NLRP3 inhibition will likely not generally predispose to infection, although it could increase risk to a small number of specific infections for which NLRP3 plays a non-redundant role in pathogen control, such as *Candida albicans*.^36^ It is also possible that blocking all pathways downstream of NLRP3 (i.e. IL-1, IL-18 and pyroptosis) could provide a beneficial niche for a pathogen sensed by this pathway.

A number of studies have investigated NLRP3 in cancer, with reports of both anti-carcinogenic and pro-tumorigenic roles for NLRP3.^37^ For example, in a recent report, murine *Nlrp3* deficiency increased metastatic foci in the liver in a model of colorectal cancer metastasis.^38^ Another report linked NLRP3 expression and caspase-1 activation to glucocorticoid resistance in leukemia cells,^39^ suggesting that blocking NLRP3 and caspase-1 activation in the latter context would be beneficial. Clearly the function of NLRP3 needs further research, especially when considering the potential long-term use of an NLRP3 inhibitor in patients with chronic diseases, but such an approach represents a new and exciting way to treat inflammation-driven diseases.

As NLRP3 activation is associated with many metabolic diseases that only develop later in life, NLRP3 blockade in older individuals may present more health benefits than risks. Given the evidence that long-term inflammation is linked with age-related decline in health^40^ and that NLRP3 may be a driving force in this process,^12^ it is tempting to speculate that NLRP3 inhibition could be a useful clinical strategy for prolonging healthspan.

## Future research

Although huge advances have been made in our understanding of the role of NLRP3 in health and disease, the basic mechanism of NLRP3 activation is still incompletely characterized.^3^ On a molecular level, understanding the NLRP3 inflammasome has been hampered by a lack of protein structural information. The structures of ASC and caspase-1 are available, but for NLRP3, the Pyrin domain is the only domain to have been solved.^41^ Structure solution for full-length NLRP3 would provide valuable insight into its mechanism of auto-inhibition, oligomerization and interaction with ASC. Accurate modeling of the entire inflammasome complex would also aid the design of NLRP3 inhibitors.

There is currently a paucity of data on the function of NLRP3 in homeostasis in healthy animals, as until now, the field’s focus has been the pathological roles of NLRP3 in disease. It will be important to further explore how NLRP3 maintains homeostasis in health, for example, the mechanisms by which NLRP3 maintains gut microbiota and regulates triglyceride metabolism, to understand the possible risks of NLRP3 inhibitory therapy in human patients. It will also be of great interest to determine whether NLRP3 blockade in human disease can reproduce the protection offered by *Nlrp3* deficiency in murine models of disease.

## Conclusion: a model for the physiological role of NLRP3

**Figure 3:**
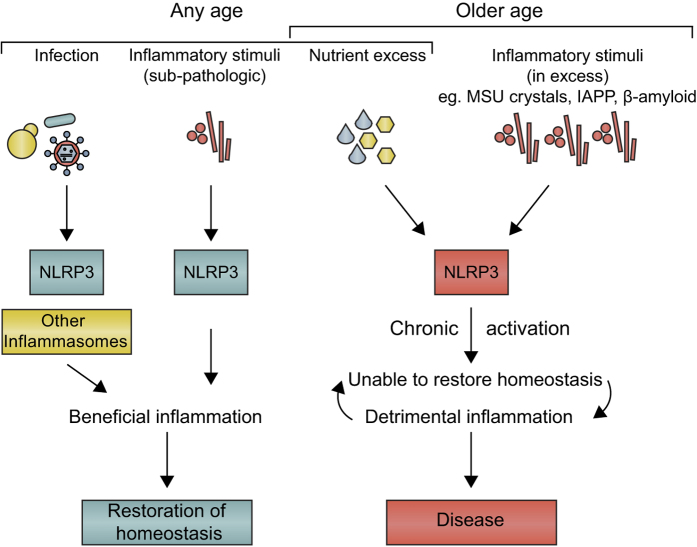
Model of the physiological role of NLRP3. NLRP3 function may be largely redundant during infection, with other inflammasomes also able to provide defense. NLRP3-dependent inflammation contributes to the restoration of homeostasis when the provoking stimuli (e.g. products of damaged tissue, including crystals) are present at low levels. In cases of excess (e.g. in response to nutrients in a high-fat diet, or high levels of deposition of noxious stimuli such as MSU crystals, or protein aggregates such as *β*-amyloid or IAPP), however, NLRP3 drives chronic inflammation, which is pathological. These events are more likely to occur with ageing, which is associated with the accumulation of such substances. Such a pathological function for NLRP3 would likely not be influenced by negative selection during evolution, as reproductive success would not be sufficiently affected. The issue in essence may therefore be one of dose—NLRP3 sensing low dose in youth being beneficial, high dose in age being detrimental.

In summary, we propose the following model for NLRP3 function in homeostasis and disease. We suggest that a primary role of NLRP3 at any age is to sense noxious stimuli that accumulate, with NLRP3 driving inflammation to facilitate their clearance (Figure 3). These factors might accumulate in the course of normal tissue damage, and the NLRP3 response would be self-limiting as the clearance succeeds. However, with age (or indeed earlier in life if there is nutrient excess), NLRP3 becomes pathologic either because of too strong a response or because of the over-activation of macrophages due to the chronic presence of excessive amounts of substances such as *β*-amyloid or MSU crystals. Another primary function of NLRP3 may be in infection, but this has evolved to be largely redundant with other inflammasomes or inflammasome-independent mechanisms of IL-1 production,^42^ likely due to the selection pressure exerted by pathogens attempting to subvert NLRP3. NLRP3 may therefore prove to be an ideal therapeutic target for major pathologies that continue to afflict humanity. As with social interactions between humans, the ageing process might give rise to irritation, NLRP3 in effect becoming the ‘grumpy old man’ of inflammation, irritated by excesses and driving diseases that are ultimately self-defeating. Reining in this behavior might, therefore, bring substantial benefits to all concerned.
